# Compliance with Specialist Referral for Increased Cancer Risk in Low-Resource Settings: In-Person vs. Telehealth Options

**DOI:** 10.3390/cancers15102775

**Published:** 2023-05-16

**Authors:** James Nguyen, Thair Takesh, Negah Parsangi, Bofan Song, Rongguang Liang, Petra Wilder-Smith

**Affiliations:** 1 Beckman Laser Institute and Medical Clinic, University of California Irvine School of Medicine, Irvine, CA 92612, USA; 2 College of Optical Sciences, University of Arizona, Tucson, AZ 85721, USA

**Keywords:** oral cancer, telehealth, low-resource settings, specialist referral, referral compliance

## Abstract

**Simple Summary:**

Most individuals are diagnosed after systemic spread of oral cancer, when treatment options and outcomes remain poor. Poor screening accuracy and lack of specialist access constitute critical barriers to improving outcomes, especially with high-risk individuals who typically have low resources and poor access to health care. The goal of this study was to investigate the impact on referral compliance in 60 subjects who had chosen either a novel in-home telehealth-specialist visit or a conventional in-person specialist visit. After 6 months, 30% of subjects had completed an in-person visit, vs. 83% who had completed a telehealth visit. Overall, 72.5% of subjects who had chosen a remote first specialist visit had received diagnosis and a treatment plan by the study end, vs. 25% of individuals in the in-person specialist group. A two-step approach that uses telehealth to overcome barriers may improve specialist referral compliance in low-resource individuals with increased OC risk.

**Abstract:**

Efforts are underway to improve the accuracy of non-specialist screening for oral cancer (OC) risk, yet better screening will only translate into improved outcomes if at-risk individuals comply with specialist referral. Most individuals from low-resource, minority, and underserved (LRMU) populations fail to complete a specialist referral for OC risk. The goal was to evaluate the impact of a novel approach on specialist referral compliance in individuals with a positive OC risk screening outcome. A total of 60 LRMU subjects who had screened positive for increased OC risk were recruited and given the choice of referral for an in-person (20 subjects) or a telehealth (40 subjects) specialist visit. Referral compliance was tracked weekly over 6 months. Compliance was 30% in the in-person group, and 83% in the telehealth group. Approximately 83–85% of subjects from both groups who had complied with the first specialist referral complied with a second follow-up in-person specialist visit. Overall, 72.5% of subjects who had chosen a remote first specialist visit had entered into the continuum of care by the study end, vs. 25% of individuals in the in-person specialist group. A two-step approach that uses telehealth to overcome barriers may improve specialist referral compliance in LRMU individuals with increased OC risk.

## 1. Introduction

Compliance with specialist referral is significantly lower in individuals from low resource, minority, and underserved (LRMU) populations with increased oral cancer risk (OC) than in other persons [1,2,3,4]. Moreover, individuals from these populations typically do not have access to routine and regular screening for OC risk [1,2,3,4]. Because disease stage at time of diagnosis and delay in treatment are primary predictors of OC outcomes [5,6,7,8,9], the burden of OC is inequitably and disproportionately worse in individuals from LRMU populations than in others [9,10,11,12]. Poor specialist referral compliance rates have been linked to barriers such as distance from the specialist’s office, fear of the unknown and of hospitals or academic settings, language barriers, workplace responsibilities, childcare and eldercare needs, and out-of-pocket costs [13,14]. Typical compliance rates with specialist referral for increased OC risk in individuals from low-resource settings (LRS) are poor, approximating 28.8–55% [15,16,17,18]. Other medical specialties report similarly low compliance rates, including surgical oncology (55%) [19], cardiac rehabilitation (33%) [20], emergency room (28%) [21], dentist (18%) [22] and general referrals (63%) [23]. Similar to OC, outcomes for many of these conditions are significantly and inequitably worse in individuals from LRMU populations than others [12,24,25,26].

Telehealth provides a potential avenue for addressing many of the critical barriers to specialist referral compliance in LRS. In-home telehealth visits overcome the need to travel to an unknown and intimidating location, reduce time taken off from childcare, eldercare, and the workplace, allow relatives or friends to assist with language barriers and provide emotional and practical support, while also facilitating a streamlined workflow for specialists. This approach has been investigated and adopted as a feasible alternative for in-person specialist consultations in many areas of medicine, especially since the onset of the COVID-19 epidemic [4,27,28,29,30,31,32,33,34]. In a study comparing the effectiveness of physical self-regulation for orofacial pains via in-person and telehealth, researchers found that patients were more likely to begin and complete self-regulation when it was offered via telehealth vs. in person [35]. In another study, researchers found that, in pediatric patients, the adjusted odds of referral completion were increased 3-fold when parents communicated remotely with the specialist prior to the in-person visit compared with those who had not [36]. 

Unfortunately, adoption of this promising tool has lagged in the field of oral health. One extensive systematic review of systematic reviews of teledentistry that included 817 citations and involved >7000 participants found that, if telehealth was employed at all, asynchronous communication was used for the majority of diagnostic/screening outcomes, despite the many limitations of this indirect form of communication [37]. It is predominantly in the field of community-based screening for OC risk that telehealth has been applied to date [38,39,40,41,42,43,44].

The objective of this study was to compare patient compliance with in-person vs. telehealth specialist referral for increased OC risk, and to identify the effect of the initial consultation format on subsequent entry into the care continuum. The long-term goal is to improve specialist referral compliance for individuals in LRS, to ensure better and more equitable health outcomes. 

## 2. Materials and Methods

This project was conducted in full compliance with University of California Irvine’s IRB-approved protocol #2002-2805. Written informed consent was obtained from all subjects involved in the study, all of whom completed the study in full compliance with the approved protocol.

### 2.1. Subjects

Sixty subjects who had screened positive for increased OC risk at Concorde College of Dental Hygiene in Garden Grove, CA, West Coast University Dental Hygiene Clinic in Anaheim, CA, and the University of California, Irvine’s Clinics were recruited. Subject demographics are shown in Table 1.

### 2.2. Protocol

After providing informed written consent at the end of their screening visit, subjects were given the choice between two different types of referral to an oral medicine specialist. They were free to opt for a conventional referral to an in-person specialist, whose office was located approximately 30 miles from the community clinic where the OC screening had been completed. Alternatively, subjects were able to choose to complete a telehealth in-home visit with the same oral medicine specialist. Subjects were told that either form of specialist visit would not incur any costs for them. Twenty individuals chose the in-person specialist referral, and forty opted for a telehealth visit. Those individuals who expressed a preference for an in-person visit were offered—at no cost—assistance with transportation, childcare, and eldercare. They were informed that an interpreter would be available to accompany them to the in-person specialist appointment, and that they would be permitted to bring one support person such as a family member or friend with them to the appointment. Individuals in the remote-visit group were provided with a very simple, very low-cost prototype smartphone-based oral telehealth system, a take-home simple instruction brochure in their language of choice, and a pre-paid return envelope for the telehealth system. They were also shown how to use the system, and the telehealth-specialist visit was scheduled. These subjects were also offered the services of an interpreter, who was scheduled to be available in the specialist’s office at the time of the telehealth visit. 

Before the remote specialist appointment, the patient read the instructions for the visit, completed a test run of the telehealth system, and applied the single-use sterile camera sheath to the intra-oral camera wand. During the telehealth visit, the patient moved the intra-oral camera wand around the oral cavity according to instructions over the smartphone from the remote oral-medicine specialist, thus completing a full 8-point visual examination of the mouth. After ending the telehealth visit, the patient repackaged the intra-oral camera and smartphone platform into the prepaid return envelope and mailed it back to the study coordinator. In all participants, referral compliance was tracked weekly over 6 months, using text messages or phone calls from the study coordinator. Patients who were non-compliant with the scheduled specialist visit received a monthly text message or phone call reminder encouraging them to do so and reiterating details of the scheduling and visit protocol. 

All subjects who had complied with the first specialist visit were subsequently scheduled for a follow-up in-person specialist visit, either for a biopsy or for follow-up, to ensure that the lesion in question had resolved satisfactorily. Again, all subjects were offered—at no cost—assistance with transportation, childcare, and eldercare. They were informed that they were permitted to bring one support person with them to the appointment, and that an interpreter would be available to assist them in their preferred language during the office visit. In all participants, referral compliance was tracked weekly over 3 months using text messages or phone calls from the study coordinator. Patients who were non-compliant with this follow-up in-person specialist visit received a monthly text message or phone call reminder encouraging them to do so and reiterating details of the scheduling and visit protocol. 

### 2.3. Telehealth System

**Figure 1:**
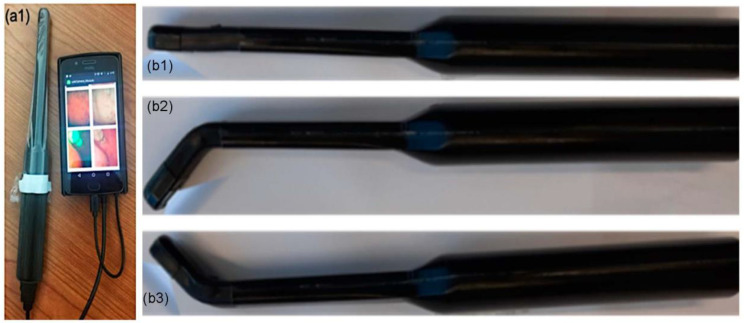
Intraoral imaging probe with disposable sheath in place, shown plugged into phone charging port (**a1**); (**b1**–**b3**) demonstrate the flexibility of the intra-oral probe.

The HIPAA-compliant prototype telehealth system implemented in this study consisted of two components, in addition to a smartphone. The first element was an intra-oral imaging probe, which the user plugged into the smartphone using the charger port. It was covered by a disposable sterile sheath prior to intra-oral use. Patients received two sheaths at the same time that they received the telehealth system, and were shown how to apply it by the study coordinator at that time. They were asked to use one sheath to practice placement at home, and to retain the second one for the actual telehealth visit. The second component of the telehealth system was a prototype bridging app, which was downloaded onto a smartphone. This app automatically connected to the Zoom telehealth app, which was also downloaded onto the patient’s phone. Figure 1(a1) shows the flexible intra-oral probe, which could be bent to access all areas of the oral cavity including the oropharynx and the base of the tongue (Figure 1(b1–b3)). The flexible zone positioned strategically in the imaging wand’s neck could be bent bi-directionally. Combined with the probe’s large focal range, this allowed for unique imaging access to all areas of the mouth, including the tonsillar pillars and floor of the mouth, as well as the posterior buccal and palatal regions, whilst minimizing contact with the oral tissues and avoiding activation of the gag reflex which commonly presents a challenge to intra-oral imaging. The telehealth app consisted of two components: (1) an app that was downloaded onto the patient’s smartphone to connect the imaging probe to the phone, allowing the patient to view the real-time image directly on their phone screen; and (2) a partner app for the remote specialist’s computer, which enabled them to view the oral cavity synchronously through the secure telehealth Zoom app. 

**Figure 2:**
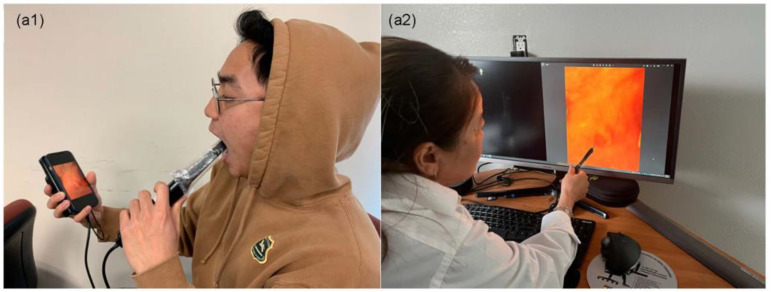
(**a1**) Subject using the intra-oral camera and smartphone system for synchronous intra-oral examination by the remote specialist. (**a2**) Clinician performing remote synchronous high-resolution intra-oral examination.

To facilitate remote oral examination, the app was installed on the remote specialist’s computer and subject’s smartphone prior to the remote visit. This app was designed to bridge seamlessly with the Zoom telehealth app. In preparation for the telehealth call, the patient simply plugged the probe into the phone charger port. Once the remote specialist called the patient using Zoom, the patient simply clicked on the telehealth icon to automatically pick up the call, initiate the connection between their phone and the probe, and share their screen with the specialist. The specialist verbally guided the patient on how to place the probe to view the regions of interest (Figure 2(a1,a2)). The specialist was also able to record still or video images, just as they would during a regular Zoom telehealth call.

At the moment when subjects in the “remote specialist” group were provided with the telehealth system, they were able to choose whether they would like to use their own phone to operate the platform, or whether they would prefer to use a simple loaner smartphone. This step was incorporated into the study protocol because we felt that some subjects might feel uncomfortable about downloading the telehealth and zoom apps onto their personal mobile phones. The loaner smartphone was a simple Android device that was disabled for all non-study use and was fitted with a tracker in case it was misplaced. 

### 2.4. Statistical Analysis

Statistical analysis was conducted using the GraphPad Prism software (GraphPad Software, Inc. Boston, MA 02110). A Fisher exact test was used to compare the proportions of subjects who showed compliance, with *p* < 0.05 considered as the level of significance.

## 3. Results

The two study groups evidenced comparable age ranges and means, and similar demographics, apart from gender distribution. 

All telehealth visits were completed in their entirety. All subjects were able to operate the telehealth platform successfully, and specialists were able to complete a full intra-oral inspection by directing the patients over the smartphone how and where to image with the probe. All telehealth platforms were returned to the study coordinators using the pre-paid, addressed envelopes that had been provided to subjects.

Overall, 29 out of the 60 subjects enrolled in this study complied with initial specialist referral within 3 months, with compliance increasing to 39 of the 60 subjects after 6 months (Table 2). The 3-and 6-month compliance rates differed significantly between the two groups, with 67.5% of individuals who had requested a telehealth visit complying within 3 months and 82.5% within 6 months, whereas only 10% of individuals who had selected the in-person option attended the specialist appointment within 3 months (*p* < 0.0001), and 30% within 6 months (*p* < 0.0001). 

Of the 39 individuals who attended the initial specialist visit, 34 complied with the follow-up in-person specialist appointment within 3 months. Amongst the subjects who had complied with a remote first specialist visit, 84.8% attended the in-person specialist follow-up appointment, and in the group which had undergone an in-person first specialist visit, 83.3% completed the in-person follow-up appointment (*p* > 0.9999).

## 4. Discussion

The overall goal of this pilot study was to evaluate the impact of telehealth on improving access to the continuum of care in individuals from low-resource settings who require an oral-cancer specialist visit. Specifically, two outcomes were assessed: (1) compliance with an initial specialist visit for assessment of cancer risk, and (2) compliance with a subsequent in-person specialist visit for a biopsy, follow-up and/or entry into the continuum of care. Our overall hypothesis was that providing a remote option for the first specialist visit would overcome many of the critical barriers to compliance, and that getting to know and trust the specialist remotely would support compliance with the second in-person specialist visit.

Subjects were allocated to the in-person or remote group based on their stated preference. Concerns that this might result in a much younger population in the telehealth group were unfounded, with similar age in each group. Moreover, twice as many individuals selected the telehealth option vs. the in-person specialist visit. This finding supports the premise that telehealth can be implemented, and indeed is welcomed, in all sectors of low-resource populations, independent of age or other demographics. This finding is supported by a broader analysis of telehealth adoption. For example, a *New York Times* (*NYT*) article reports that nine million people under Medicare alone used telemedicine services during the early months of the [COVID-19] crisis [45]. Early data does not show wide variations in use by race or ethnicity [45]. In addition to federal spending through Medicare, nearly USD 4 billion was billed nationally for telehealth visits during March and April, compared to less than USD 60 million for the same two months of 2019 [46].

A novel telehealth platform was used that was specifically designed for use in low-resource settings. Because the system was delivered to patients during their screening visit for oral cancer risk in a community clinic, they were able to receive an in-person demonstration on set-up and operation before taking the platform home with them. This demonstration lasted less than 5 min, underlining the custom, simple and intuitive design of the system specifically for a population accustomed to operating a mobile phone and using simple apps, but with no technical skills beyond this level. Thus, the investigators were able to confirm that the system and its mode of operation were indeed well-suited to this study’s target population. Since the telehealth call was initiated and controlled by the specialist, all the patients had to do was to accept the secure Zoom call on their phone, and then scan the imaging wand around their mouth based on the specialist’s instructions on the linked smartphone. One other early study using this system also reported successful adoption and implementation by individuals in low-resource settings [29].

Overall compliance with the first specialist visit by all subjects in both groups combined measured only 48.3% after 3 months, and 65% after 6 months. It was interesting to note that the increase in referral compliance between month 3 and month 6 for both groups was very similar, measuring approximately 20%. Unfortunately, there is little information in the literature regarding the timeline for specialist referral compliance, but there are many studies investigating the cause for delay in referral compliance due to administrative bottlenecks and poor specialist availability. It would be interesting and constructive to obtain a better understanding of the triggers that advance the decision-making process leading to referral compliance. 

Entry into the continuum of care by all subjects averaged a mere 56.7%. For the subjects who had chosen to complete their first specialist visit in person, in accordance with common usage, only 25% of subjects had entered into the continuum of care by study completion, whereas 72.5% of subjects in the telehealth group had progressed into the continuum of care. Study results show that patients from either group who complied with the first referral visit were equally likely to comply with the second specialist appointment. These findings underline the importance and potential impact of telehealth in improving access to specialist care, by overcoming barriers to the first specialist visit. As oral cancer outcomes are primarily determined by cancer stage at time of diagnosis, and an increase in the time up to treatment of as little as 2 months significantly increases risk of death [5,10], better compliance with specialist referral can serve as a powerful tool to address the inequitably high morbidity and mortality that are experienced by individuals in low-resource settings [6,7,11,47,48]. Referral compliance was greatly improved in the telehealth group, with significantly better referral compliance rates at 3 months and 6 months, and an almost three-times-greater rate of entry than the in-person group into the care continuum by the study end. These findings support our hypothesis that telehealth visits may indeed overcome barriers to specialist visits and entry into the care continuum, by overcoming fear of the unknown, by demonstrating effective measures to overcome language barriers, and, most importantly perhaps, by establishing a personal relationship of trust and care between the clinician and the patient. While there is little information on this concept in the literature, a few studies have reported a considerable increase in specialist referral compliance when the shepherding process was carried out using telehealth [35,36,49]. Perhaps those findings support our hypothesis that introducing a face and personal interactions into the process of undergoing referral builds trust and familiarity, thus reinforcing the will to comply and overcoming the barriers posed by fear of the unknown.

This pilot study had several weaknesses. Subjects were permitted to select their preferred group allocation, which may have led to bias in their compliance and behavior. A classical randomized allocation process would have been preferrable in some ways. Because this is a pilot study, the investigators chose to let subjects select their own preferred form of specialist visit because this provision of choice parallels current practice in many areas of medicine. Patients are routinely offered the choice between in-person and telehealth visits for much of their medical care. Moreover, the study design conforms to telehealth implementation protocols as we envisage them in future routine use in community clinics. Larger randomized studies—whose scope exceeds that of this first pilot study—are needed in future, to address this matter. 

The study added considerable impetus to ensuring referral compliance in both groups through its monthly reminder communications to both groups, and weekly compliance checks. Thus, the study outcomes for both groups do not fully represent the real-life situation and may be overly optimistic, especially as considerable assistance with the costs and logistics of the in-person specialist visits was provided. It should also be noted that patients in the in-person group may have experienced visit fatigue or frustration with multiple visits, which may have added reluctance to compliance, but which also reflects the real-life situation of an in-person specialist pathway. Finally, the effect on telehealth success of mailing the system and instructions to the patient without providing in-person training (albeit less than 5 min in this study) needs to be elucidated. Larger studies are now underway to address these shortcomings and gather more comprehensive data.

## 5. Conclusions

Specialist referral compliance and entry into the care continuum for individuals with increased OC risk from low-resource settings may be improved using a novel oral telehealth platform. 

## Figures and Tables

**Table 1:** Study Population Demographics.

	Remote Specialist Visit	In-Person Specialist Visit
Mean Age (Years)	58	56
Age Range (Years)	39–82	42–79
Median Age (Years)	51	54
Asian	14	6
Pacific Islander	1	0
White non-Hispanic	6	5
White Hispanic	14	7
African American	2	1
Mixed Race	3	1
Female	21	14
Male	19	6
**Total**	**40**	**20**

**Table 2:** Compliance with Initial and Follow-up Specialist Visits and Entry into Care Continuum.

	Remote Specialist (*n*, %)	In-Person Specialist (*n*, %)	Remote vs. In-Person Comparison: (Sig; *p*-value)	Total (*n*, %)
Compliance with Specialist Referral within 3 Months	27/40 (67.5%)	2/20 (10%)	Yes; *p* ≤ 0.0001	29/60 (48.3%)
Compliance with Specialist Referral within 6 Months	33/40 (82.5%)	6/20 (30%)	Yes; *p* ≤ 0.0001	39/60 (65%)
Compliance with In-Person Specialist Follow-up Visit for Those Who Completed Initial Specialist Visit, within 3 Months after Initial Visit	29/33 (84.8%)	5/6 (83.3%)	No; *p* ≥ 0.9999	34/39 (87.2%)
Entry into Care Continuum Out of All Subjects in each Group	29/40 (72.5%)	5/20 (25%)	Yes; *p* = 0.0008	34/60 (56.7%)

## Data Availability

Data supporting reported results may be available upon request.
